# Hydrophobically Grafted Pullulan Nanocarriers for Percutaneous Delivery: Preparation and Preliminary In Vitro Characterisation

**DOI:** 10.3390/polym13172852

**Published:** 2021-08-25

**Authors:** Mohammad F. Bostanudin, Eugen Barbu, Kai Bin Liew

**Affiliations:** 1 College of Pharmacy, Al Ain University, Abu Dhabi 112612, United Arab Emirates; 2 School of Pharmacy and Biomedical Sciences, University of Portsmouth, St Michael’s Building, White Swan Road, Portsmouth PO1 2DT, UK; eugen.barbu@port.ac.uk; 3 Faculty of Pharmacy, University of Cyberjaya, Cyberjaya 63000, Malaysia; liewkaibin@cyberjaya.edu.my

**Keywords:** biopolymers, nanomaterials, polymer synthesis, pullulan, percutaneous

## Abstract

Polymeric colloidal nanocarriers formulated from hydrophobically grafted carbohydrates have been the subject of intensive research due to their potential to increase the percutaneous penetration of hydrophilic actives. To this goal, a series of hydrophobically grafted pullulan (BMO-PUL) derivatives with varying degree of grafting (5–64%) was prepared through functionalisation with 2-(butoxymethyl)oxirane. The results demonstrated that monodispersed BMO-PUL nanocarriers (size range 125–185 nm) could be easily prepared via nanoprecipitation; they exhibit close-to-spherical morphology and adequate stability at physiologically relevant pH. The critical micellar concentration of BMO-PUL was found to be inversely proportional to their molecular weight (Mw) and degree of grafting (DG), with values of 60 mg/L and 40 mg/L for DG of 12.6% and 33.8%, respectively. The polymeric nanocarriers were loaded with the low Mw hydrophilic active α-arbutin (16% loading), and the release of this active was studied at varying pH values (5 and 7), with a slightly faster release observed in acidic conditions; the release profiles can be best described by a first-order kinetic model. In vitro investigations of BMO-PUL nanocarriers (concentration range 0.1–4 mg/mL) using immortalised skin human keratinocytes cells (HaCaT) evidenced their lack of toxicity, with more than 85% cell viability after 24 h. A four-fold enhance in arbutin permeation through HaCaT monolayers was recorded when the active was encapsulated within the BMO-PUL nanocarriers. Altogether, the results obtained from the in vitro studies highlighted the potential of BMO-PUL nanocarriers for percutaneous delivery applications, which would warrant further investigation in vivo.

## 1. Introduction

The development of percutaneous drug delivery has aroused enormous interest owing to its potential to avoid first-pass effect and gastrointestinal enzyme-catalysed degradation. Nevertheless, actual progress in the delivery of numerous water-soluble actives has been restrained by their inadequate percutaneous penetration due to the nature of the skin lipid layer that has a high affinity for hydrophobic, ionised, and low molecular weight compounds [1,2]. In an effort to increase the permeation of these actives across the skin, various approaches such as physical (i.e., iontophoresis, magnetophoresis, microneedle, etc.) and chemical (i.e., alcohol, amides, esters, etc.) enhancers have been considered [3]. Despite recent technological advances, harmful side effects such as skin irritation reactions combined with low drug permeability continue to pose major challenges for an effective percutaneous delivery [4,5]. In an alternative approach that shows promising results, ongoing research actively aims at the polymeric colloidal nanocarriers development for percutaneous drug delivery [6,7,8].

Among the various naturally occurring polymers with potential in drug delivery, of particular interest is pullulan, a widely bioavailable and water-soluble polysaccharide synthesised by *Aureobasidium pullulans* fungus [9]. Well-known for its biodegradability and lack of toxicity, pullulan has been used mainly in the pharmaceutical and food industries [10]. It has been investigated for skin and percutaneous administration, with results suggesting its potential in treating skin wounds and in enhancing percutaneous drug delivery [11,12,13]. The biogenic synthesis pathway of nanocarriers is considered inexpensive, nontoxic, and eco-friendly [14,15]. Biogenic pullulan nanocarriers have shown potential in a variety of applications, including antimicrobial [16], anticancer [17], and antibacterial activities [18]. Pullulan-based nanocarriers have also demonstrated their ability in percutaneous protein delivery [19]. Pullulan possesses functional groups that facilitate a range of chemical modifications that can make it more amenable for use in various applications [20]; for example, when modified into a material with a hydrophobic character, it can spontaneously self-assemble into nanocarriers with a high loading capacity and low critical micellar concentration (CMC) [21,22]. Additional benefits of amphiphilic nanocarriers such as decreased drug cytotoxicity, prolonged blood circulation time, and enhanced permeation make these systems very promising for percutaneous delivery [23,24,25,26,27,28].

One approach to introduce a degree of hydrophobic character to polysaccharides is alkylation, which results in modified physicochemical properties affecting the membrane permeation ability [29] and ultimately the bioactivity of the whole macromolecule [21,30,31]. Different reagents, including epoxide derivatives such as 2-(butoxymethyl)oxirane, can be used to add alkyl groups to hydrophilic polysaccharides. The epoxide ring opening, which is catalysed by a base, transforms it to a hydrophobic alkylglycerol, which may then be grafted onto polysaccharide-free hydroxyl groups to produce hydrophobically grafted polysaccharides [32]. α-arbutin, a hydrophilic antioxidant and topical tyrosinase inhibitor, was used as a model drug following studies reporting its poor skin permeability due to the high hydrophilicity of the compound despite being considered as one of the most efficient whitening agents [33,34,35]. Different studies have lately sought to study the efficient percutaneous administration of α-arbutin, both topically and transdermally, to enhance its skin permeability due to its wide variety of uses in various applications [36,37].

To our knowledge, there have been no studies investigating the potential for the percutaneous delivery of α-arbutin using nanocarriers formulated from hydrophobically grafted pullulan with 2-(butoxymethyl)oxirane. Investigating the hypothesis that nanoformulations prepared from hydrophobically grafted polymers can provide improved drug carrier characteristics that are suitable for percutaneous delivery, we present here the synthesis and characterisation of hydrophobically grafted pullulan (BMO-PUL) that was grafted with hydrophobic alkylglycerol, 2-(butoxymethyl)oxirane, through a S_N_2 nucleophilic substitution reaction under aqueous alkaline conditions. The formulation and characterisation of their corresponding self-assembled nanocarriers that were formulated via nanoprecipitation and loaded with α-arbutin are presented. The loading ability, release profiles, and stability of the nanocarriers at physiologically pertinent conditions, as well as the results of the in vitro investigation subject to nanoparticle interactions with immortalised human skin keratinocyte cells (HaCaT) as regards cytotoxicity and membrane permeability are also discussed.

## 2. Materials and Methods

### 2.1. Materials

Pullulan (Mw 100 kDa), 2-(butoxymethyl)oxirane (BMO; analytical grade 95%), α-arbutin, sodium hydroxide (NaOH), phosphate-buffered saline (PBS), phosphate-citrate buffer (PCB), Dulbecco′s Modified Eagle′s Medium (DMEM), Hank′s balanced salt solution (HBSS), foetal bovine serum (FBS), rat tail collagen, and trypsin-EDTA were obtained from Sigma-Aldrich Chemie GmbH (Steinheim, Germany). Dimethyl sulfoxide (DMSO; HPLC grade ≥ 99.9%), diethyl ether (analytical grade), hydrochloric acid (HCl), H_2_O (HPLC grade), and methanol (HPLC grade ≥ 99.9%), were supplied by Fisher Scientific (Loughborough, UK).

### 2.2. Preparation of Grafted Pullulan (BMO-PUL) Derivatives

The synthetic method was adapted from the literature [38], as follows: to an aqueous pullulan solution (0.5 g; 3.05 mmol; 45 mL), a solution of sodium hydroxide (33% *w*/*v*; ~0.5 mL) was incrementally introduced under stirring to alkalinise (pH 14) the mixture; the stirring was maintained for 2 h prior to storage (−20 °C) for 5 days. Following thawing at 25 °C, 2-(butoxymethyl)oxirane (3.05 mmol, 0.44 mL) was introduced dropwise under vigorous magnetic stirring (LMS-1003, Daihan Labtech Co., Ltd., Namyangju, Korea) and further agitated at 45 °C for another 16 h (under N_2_ atmosphere); the process was repeated using different concentrations (15.25 mmol, 2.18 mL and 24.40 mmol, 3.49 mL and 57.95 mmol, 8.29 mL and 91.5 mmol, and 13.09 mL) to investigate the influence of reagent (BMO) concentration upon the degree of grafting (DG). The mixture was then neutralised by hydrochloric acid (10% *v*/*v*) and purified by washing with diethyl ether (×3) to exclude water-insoluble impurities. The mixture was subsequently purified by dialysis (molecular weight cut-off 12–14 kDa) (10 L ion-free H_2_O; 9 changes over 72 h) and lyophilised (VirTis Sentry 2.0, SP Scientific, Genevac Ltd., Ipswich, UK).

### 2.3. Characterisation of Grafted Pullulan (BMO-PUL) Derivatives

Nuclear magnetic resonance (NMR) spectroscopic analysis was carried out by dint of a JEOL Eclipse 400 instrument (JEOL, Welwyn Garden City, UK) running at 400 MHz and 100 MHz for ^1^H- and ^13^C-NMR, respectively; the DG was determined from the ^1^H-NMR spectra. A Nicolet Nexus Euro Fourier-transform infrared (FTIR) spectrometer (Thermo Fisher Scientific, Hemel Hempstead, UK) fitted with a Smart Orbit diamond crystal attenuated total reflectance (ATR) was utilised to record the FTIR spectra (scans = 32, resolution = 4 cm^−1^ and wavenumber range = 4000–500 cm^−1^). Thermal analysis methods such as thermogravimetric analysis (TGA) and differential scanning calorimetry (DSC) were done on TG 209 F1 Libra (heating rate = 10 K/min, temp. range = 25 to 500 °C, and N_2_ purge flow rate = 40 mL/min) and DSC 214 Polyma (heating and cooling rate = 10 K/min, temp. range = −50 to 250 °C, and N_2_ purge flow rate = 40 mL/min) instruments (NETZSCH, Selb, Germany), respectively.

Native and BMO-PUL were investigated for their viscosity by a Gilmont GV-2100 falling-ball viscometer (Gilmont Instruments Inc., Barrington, IL, USA). An aqueous polymer solution (1% *w*/*v*) was introduced into the viscometer cylindrical tube (~10 mL) and a glass sphere (density = 2.53 g/mL) was carefully introduced. The elapsed time required for the glass sphere to fall under gravity through the samples was recorded and the viscosity was determined utilising Equation (1) (viscometer constant = 0.3 and liquid density = 1.0 g/mL):

(1) η=K (ρt − ρ) t

where

η: calculated viscosity (cp)

ρ_t_: ball density (g/mL)

ρ: liquid density (g/mL)

t: descent time (min)

K: viscometer

Molecular weights were measured on a Waters Alliance GPC-2000 gel permeation chromatography (Waters Corporation, Milford, MA, USA) fitted with a PL aquagel-OH gel column (8-µm particle size) under controlled temperature (30 °C), with refractive index detection. Eluent system of either 100% H_2_O or an 8:2 (*v*/*v*) H_2_O:DMSO mixture (HPLC grade) was employed (flow rate = 0.5 mL/min). Pullulan standards (Mws 0.6–80.5 × 10^4^ g/mol; Showa Denko, New York, NY, USA) calibration curves were utilised to estimate the molecular weight (Mw) of the grafted pullulan.

### 2.4. Formulation of Nanoparticles from Grafted Pullulan (BMO-PUL)

To ultrapure H_2_O (8 mL), a solution of BMO-PUL in dimethyl sulfoxide (2 mL) (concentration range 1, 5, and 10 mg/mL with various DG, as specified) was introduced dropwise under constant stirring. The resultant BMO-PUL nanoformulations were dialysed (molecular weight cut-off 12–14 kDa) (10 L ion-free H_2_O; 9 changes over 72 h) before being collected by freeze-drying (yields 76–83%).

### 2.5. Physical Characterisation of Grafted Pullulan (BMO-PUL) Nanoparticles

The critical micellar concentration (CMC) of BMO-PUL amphiphiles and the hydrodynamic diameter of the self-assembled carriers were measured via DLS using a Malvern ZetasizerNano ZS instrument (Malvern Instruments, Malvern, UK; 633 nm He-Ne laser; back-scattering angle of 173°; Zetasizer v7.01 software). The samples were tested in triplicate (25 °C); the NPs size was reported as the Z-average mean values (Z-av.) together with the dispersity index (DI). Changes in the scattered light intensity whilst varying the sample concentration (30–70 mg/L) were monitored to determine the CMC. The zeta (ζ) potential of the nanoparticles was figured out by electrophoretic mobility measurements (EPM) on a similar instrument; the results were processed based on Smoluchowski’s formula (Henry’s function f(ka) = 1.5).

Morphological investigation of the nanoparticles was done with a JEOL-JSM-6060LV (JEOL, Tokyo, Japan) electron scanning microscope (SEM). Sample preparation was done by placing a dispersion of lyophilised nanoparticles in ultrapure H_2_O (5 mg/mL) onto a metallic stub and dried before being coated with Au/Pd (Ar atmosphere) on a Quorum Q150RES sputter coater (Quorum Technologies Ltd., East Sussex, UK).

### 2.6. Loading and Release Studies

The nanoparticle loading capacity was examined utilising α-arbutin as a model active following loading into NPs via nanoprecipitation as follows: to a solution of BMO-PUL (DG 33.8%) in DMSO (2 mL), α-arbutin (0.5 mg) was dissolved prior to the dropwise addition into ultrapure H_2_O (8 mL) under strong stirring. The resultant nanocarriers were centrifuged (164,391× *g*; 30 min; 20 °C); the pellets were freeze-dried and collected. A Reversed-Phase (RP) HPLC (λ = 222 nm) on a Jasco LC-4000 series HPLC system (Jasco Inc., Easton, MD, USA); C18 RP column, 89:10:1 (*v*/*v*/*v*) H_2_O:MeOH:0.1 M HCl; flow rate = 1 mL/min; retention time = 5.7 min, and lower detection limit = 25 ng/mL) was utilised for the analysis of the supernatant to estimate the free α-arbutin quantity. The collected data were plotted against the calibration curves, and the loading (presented as the ratio of drug weight to the total weight of NPs) was estimated utilising Equation (2):

(2) $$\mathrm{DL}\;\left(\%\right)=\frac{\mathrm{weight}\;\mathrm{of}\;\mathrm{drug}}{\mathrm{weight}\;\mathrm{of}\;\mathrm{nanoparticles}\;}\times 100$$

The investigation of α-arbutin release was conducted by redispersing the α-arbutin-loaded BMO-PUL NPs (2 mg/mL) in either PCB (pH 5) or PBS (pH 7) medium (4 mL) and placed into Eppendorf tubes before being kept in a shaking water bath (WSB-30, Daihan Labtech Co., Ltd., Korea) under controlled temperature (37 °C). Every Eppendorf tube was removed from the water bath at predetermined time points prior to aliquot withdrawal (2 mL) from the supernatant. The analysis was performed through RP-HPLC.

### 2.7. Stability Studies

Nanoparticle stability was assessed for 90 days by redispersing (2 mg/mL) and incubating α-arbutin-loaded BMO-PUL (DG 33.8%) nanoparticles in either PCB (pH 5) or PBS (pH 7) at various temp. (either 4 or 25 °C). Any possible changes in the Z-av., ZP, and DI of the nanoparticles were analysed with a Malvern ZetasizerNano ZS.

### 2.8. Cell Culture

Immortalised human keratinocytes (HaCaT) cells were acquired from Cell Lines Service (Eppelheim, Germany). The cells (passage no. 17–25) were cultured (in Corning T25 flasks; media volume 6 mL) in DMEM (enriched with 2 mM L-glutamine, 0.1 mg/mL streptomycin, 100 U/mL penicillin, and 10% *v*/*v* foetal bovine serum) at 37 °C in a humidified ambiance with 5% CO_2_. The detachment of cells was done using trypsin-EDTA, prior to being harvested by centrifugation (120× *g*; 5 min) for 5 min.

### 2.9. Cytotoxicity Assay

α-arbutin-loaded BMO-PUL NPs suspensions in DMEM (50 μL; 0.1–4 mg/mL) were cultured (37 °C) with confluent HaCaT cells for 24 h (seeding 5 × 10^3^). The medium was then substituted with MTT (3-(4,5-dimethylthiazol-2-yl)-2,5-diphenyltetrazolium bromide) reagent (100 μL; 10% *w*/*v*) and incubated for another 3 h before being substituted by DMSO (100 μL). Initially, the MTT reagent was prepared by dissolving 3-(4,5-dimethylthiazol-2-yl)-2,5-diphenyltetrazolium bromide in a serum-free DMEM media (1 mg/mL). The plate was then analysed on a Multiskan GO microplate reader (Thermo Fisher Scientific, Waltham, MA, USA; measuring at 570 nm).

### 2.10. Permeability Studies across HaCaT Cell Monolayers

**Figure 1:**
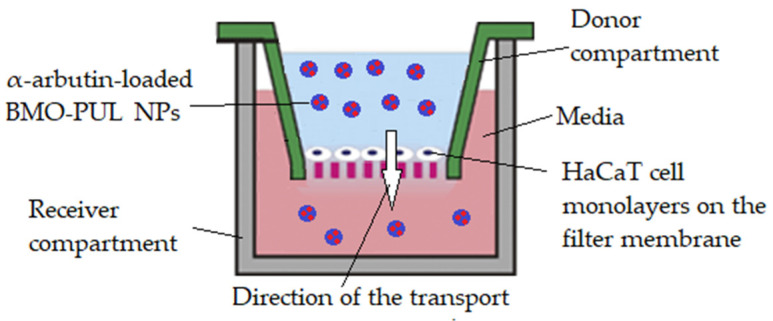
Illustration of the modified Transwell-type HaCaT model employed in these studies.

The test was adapted from a method described in the literature [39] as follows: a modified Transwell-type HaCaT model (Figure 1) was set up by introducing HBSS (200 μL), collagen (100 μL; 0.4%), DMEM (100 μL), and FBS (10% *v*/*v*) mixture into a sterile Millipore Millicell 24-well plate (Millipore, Billerica, MA, USA) prior to 30 min of incubation (5% CO_2_; 37 °C). After the collagen gel solidified, a HaCaT cell suspension was planted (5 × 10^3^ cells) and cultivated until confluency. Individual wells were filled with dispersions of α-arbutin-loaded BMO-PUL nanocarriers (200 μL; 4 mg/mL) in DMEM and the α-arbutin conc. in the receiver compartment was observed. Aliquots (100 μL) were withdrawn every 1 h from the receiver compartment throughout 5 h); they were sonicated (10 min) and ultracentrifuged (164,391× *g*; 20 min; 25 °C; X3 Beckman, Beckman Coulter, High Wycombe, UK), and the supernatant was then analysed through RP-HPLC. The experiment was repeated using free α-arbutin (200 μL; 0.5 mg/mL dissolved in DMEM) for comparison and the apparent permeability coefficient (Papp) was calculated utilising Equation (3):

(3) $$\mathrm{Papp}\;\left(\mathrm{cm}.s^{-1}\right)=\frac{\mathrm{dQ}}{\mathrm{dt}}\times \frac{1}{A\;\times \mathrm{Co}}$$

where

dQ/dt: α-arbutin flux translocated via the membrane (µg/sec),

A: filter surface area (0.33 cm^2^) and

Co: α-arbutin original mass conc. at the donor compartment (100 µg/cm^3^).

### 2.11. Data Statistical Analysis

All data were acquired from at least *n* = 3 (unless mentioned otherwise) and statistically analysed by SPSS version 22 software (SPSS Inc., Chicago, IL, USA). The statistical significance was calculated using one-way analysis of variance (ANOVA) with a *p* ≤ 0.05, unless indicated otherwise. The results were depicted as the mean ± standard deviation (SD).

## 3. Results and Discussion

**Figure 2:**
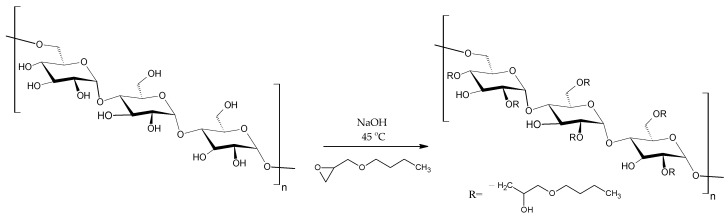
A schematic of the synthesis of grafted pullulan (BMO-PUL).

BMO-PUL derivatives of pullulan were prepared by reacting the native polysaccharide with BMO in an aqueous alkaline solution (NaOH, pH 14; Figure 2). NaOH converts the pullulan OH groups into alcoholates, which react via a nucleophilic substitution with the epoxide ring and, thus, facilitate the grafting of the hydrophobic alkyl chain [2-(butoxymethyl)oxirane] to the polysaccharide chain [38]. According to the literature, the attachment of the hydrophobic alkyl chain is expected to mainly take place at the C^6^ primary OH group, owing to its high reactivity and reduced steric hindrance [40,41,42,43].

**Figure 3:**
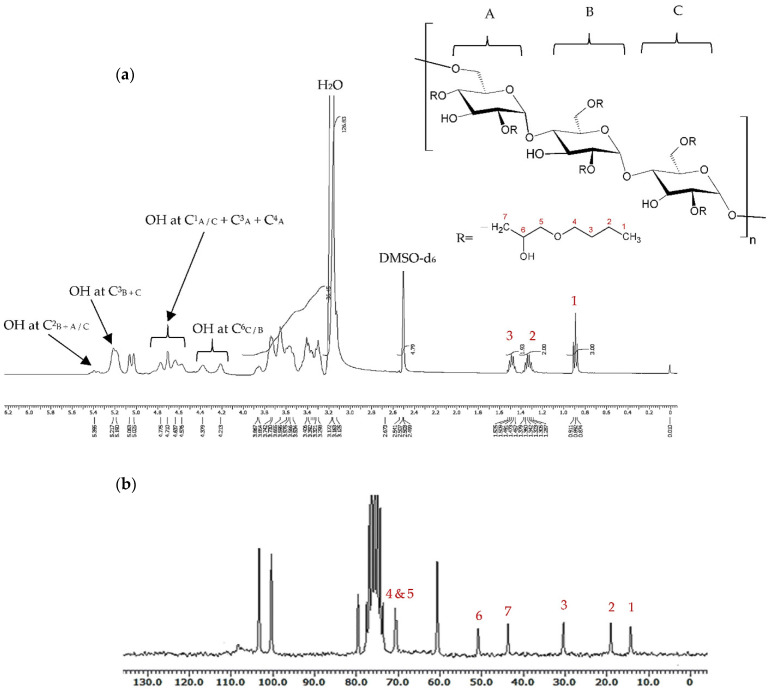
Typical ^1^H-NMR (**a**) and ^13^C-NMR (**b**) spectra of BMO-PUL in DMSO-d_6_ (7 mg/mL). The three sugar rings of BMO-PUL were ascribed as A, B, and C.

The lyophilised products were acquired as low-density, white cotton-like materials (yield 81–84%), with the chemical modification being verified by ^1^H- and ^13^C-NMR, as well as FTIR spectroscopy. The Mw was in the range 100–136 kDa, as analysed by gel permeation chromatography (GPC). In the ^1^H-NMR spectra of BMO-PUL (Figure 3a), the peaks of pullulan backbone protons appeared over the spectral range δ 3.3–5.4 ppm; 3.3–4 ppm assigned to proton signals at C^2–6^, whilst 5–5.4 ppm was assigned to anomeric protons [11]. The signals recorded at δ 0.9, δ 1.3, and δ 1.5 ppm (denoted as peaks 3, 2, and 1 in Figure 3, respectively) were indicative of the existence of the hydrophobic alkyl chain groups [38]; other protons signal assignments are presented in Figure 3a. The ^13^C-NMR spectrum concurred with the ^1^H-NMR data, with alkyl chain carbons detected at δ = 14, 19, 31, 44, 51, and 71 ppm, as displayed in Figure 3b. The ^13^C-NMR spectrum of the BMO reagent is recorded in Figure S1, Supplementary Materials. Other significant peaks in the ^13^C-NMR spectrum were depicted as follows: δ = 101–103 ppm for polysaccharide C^1,^ δ = 68–77 ppm for C^2,3,5^, δ = 80 ppm for C^4^, and δ = 61 ppm for C^6^ [41].

The degree of grafting (DG) was calculated based upon the ratio of the integral values of the alkyl chain end CH_3_ group (δ 0.9 ppm) to the glucopyranosic ring sugar protons (δ 3.3–4.0 ppm), as evidenced by the ^1^H-NMR spectra. The DG (presented as hydrophobic alkyl chain number grafted to 100 glucopyranose units in the pullulan molecule; i.e., 100% = all three possible sites in a single sugar residue were completely grafted) of BMO-PUL was calculated utilising Equation (4):

(4) $$\mathrm{DG}\;\left[\%\right]=\frac{2A\;\times 100}{3B}$$

where

DG (%): degree of grafting (hydrophobic alkyl chain number attached to 100 sugar residues),

A: integral of the peak ascribed to the alkyl chain end CH_3_ (δ 0.9 ppm).

B: integral of the signal ascribed to the glucopyranosic ring sugar protons (δ 3.3–4.0 ppm).

**Figure 4:**
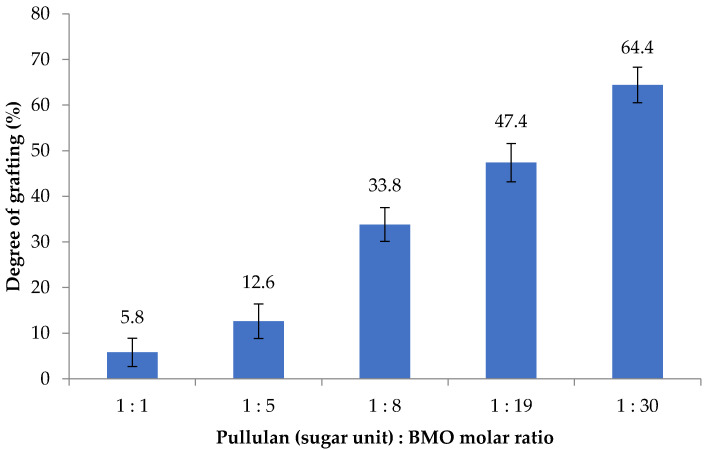
The effect of reagent ratio on the grafting degree (*n* = 3; ± SD).

The effect of BMO reagent conc. on BMO-PUL degree of grafting was also studied; as illustrated in Figure 4, the DG increased together with an increase in the reagent concentration. The calculated DG was almost linearly proportional to the molar ratio (y = 1.98x + 7.80; *r*^2^ = 0.93), indicating the potential capability of pullulan in taking in greater molar ratios than were employed to afford BMO-PUL with greater DG. The DG of BMO-PUL obtained in this study, however, was found to be lower than that of hydrophobically grafted pullulan synthesised in a nonaqueous environment (DG 47–77%; potassium tert-butoxide (KOt-Bu) as the catalyst) - albeit using a similar pullulan:BMO molar ratio. This is perhaps owing to the catalyst/base strength used in the reaction; KOt-Bu is considerably powerful than NaOH and, hence, enhanced the alcoholates reactivity to react with the opened-ring epoxide [44]. Moreover, the presence of H_2_O that may serve as a nucleophile, making itself possible to interfere with the reaction, which may also reduce the reaction rate between the alkylglycerols and pullulan [45].

**Figure 5:**
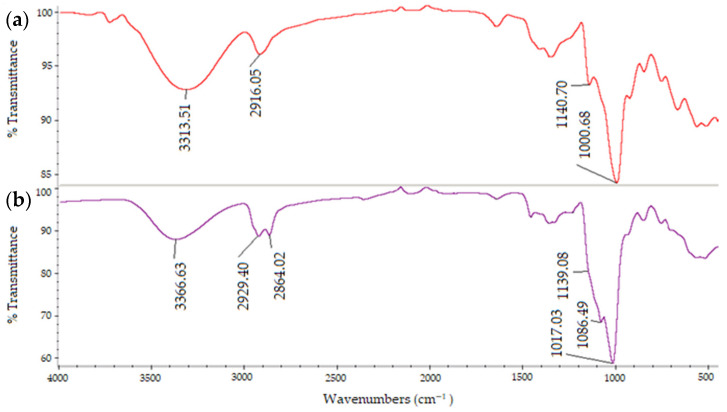
Typical FTIR-ATR spectra of a (**a**) native pullulan and (**b**) BMO-PUL.

As illustrated by the FTIR spectra results (Figure 5), the major bands of native pullulan and BMO-PUL were exhibited and assigned as follows: wide peaks at 3363–3313 cm^−1^ ascribing O–H groups stretching resulting from intra- and intermolecular hydrogen bonding, peaks at 2929–2916 cm^−1^ ascribing –CH_2_ asymmetric stretching, and peaks at 1140–1000 cm^−1^ ascribing C–O–C stretching from glycosidic bonds [46,47]. Functionalisation of pullulan with 2-(butoxymethyl)oxirane (Figure 5b) resulted in the emergence of a new C–H peaks (assigned to a –CH_3_ symmetric stretching vibration at 2864 cm^−1^) and an enhance in the ether (C–O–C) band intensity (1140–1000 cm^−1^) in which, consistent with our previous alkylglycerol modification done on pectin [38]. According to Wang et al., the enhanced peak intensity at ca. 1110 cm^−1^ is also expected owing to the stretching vibration of the linear fatty ether backbone of the alkyl chain [48]. A typical 2-(butoxymethyl)oxirane with an intact epoxy ring has peaks at ca. 915–760 cm^−1^, however, based on Figure 5, those regions were found to be relatively unchanged following the modification this could indicate the success of the epoxy ring-opening in the reaction, as well as the complete removal of unreacted reagent in the final product [49].

**Figure 6:**
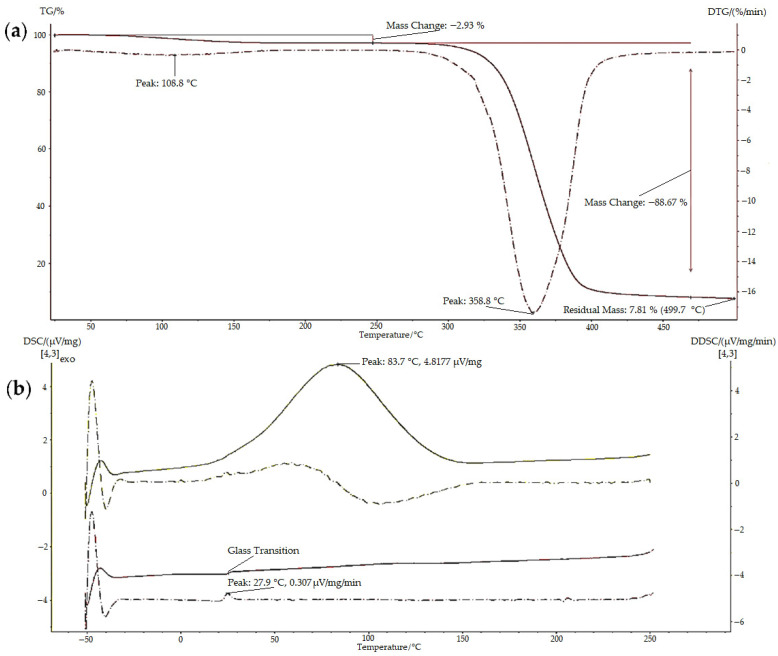
Representative thermal analysis results for BMO-PUL: (**a**) TGA thermogram: 1st derivative TG (DTG) depicted as a dotted line and (**b**) DSC curves (top: 1st run and bottom: 2nd run).

Thermal characterisation (TGA and DSC) was conducted to examine the properties, thermal transitions, and stability of the polymers when the temperature was gradually changed; examples of the results obtained are illustrated in Figure 6. The TGA thermogram of BMO-PUL (Figure 6a) showed multiple steps related to different mass loss processes. The initial mass loss step is attributed to evaporating the moisture entrapped in the materials, whereas the second step is ascribed to the thermal decomposition of the materials [50].

Table 1 shows thermal analysis data attained for some samples, and the results affirm the expected increase in the hydrophobicity of the polysaccharide following the functionalisation with 2-(butoxymethyl)oxirane. The measured H_2_O content was found to be inversely proportional to the DG (native pullulan possessed more H_2_O, >6%, compared to the BMO-PUL with values in the range 3–5%). Lower hydrogen-bonded H_2_O molecules in BMO-PUL derivatives may be attributed to enhanced hydrophobicity after the modification, which ultimately limits them from retaining additional moisture. This is in line with earlier studies on the alkylation of polysaccharides (dextran and pectin) with short-chain alkylglycerols [31,38]. BMO-PUL showed increased thermal stability compared to the native, as evidenced by the DTG peak corresponding to the decomposition of the BMO-PUL derivatives that surfaced at a greater temperature.

The polysaccharide glass transition temperature (Tg) moved to higher temperatures following the modification. The introduction of the hydrophobic alkyl chain increased the molecular structure bulkiness of the polysaccharide and led to a reduction in mobility. The alteration in BMO-PUL hydrophobicity results in the removal of hydrogen-bonded H_2_O molecules, which can serve as a plasticiser to increase polymer chain mobility and reduce intermolecular forces, leading to a lower Tg value of amorphous solids, such as the native pullulan (Table 1) [51,52]. The Tg values of pullulan discussed in the literature differ noticeably. Bizot et al. studied the Tg of pullulan and found that the value varies from 10 to 25 °C [53]. As Tg values are affected by the composition of each sample, hence, the differences recorded are attributed to batch-to-batch variations that exist in terms of composition, molecular weight, and H_2_O content [54].

**Figure 7:**
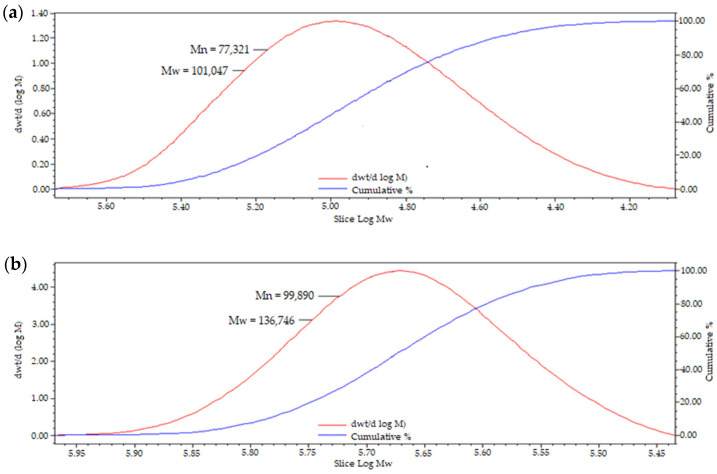
Representative GPC chromatogram of (**a**) native pullulan and (**b**) BMO-PUL (DG 33.8%).

Gel permeation chromatography was utilised to study the Mw of pullulan following the modification, and it has been found that functionalisation with 2-(butoxymethyl)oxirane to afford hydrophobically grafted BMO-PUL yielded in an enhance in the Mw (Table 2), which validates the successful attachment of the hydrophobic alkyl chain to the pullulan backbone. Dispersity index (DI) values that are close to one suggested a narrowly dispersed polymer distribution and homogenous branching. GPC chromatogram of BMO-PUL is exemplified in Figure 7.

Investigation on the viscosity of pullulan biopolymer following modification was done, and the results are exemplified in Table 3. A decrease in the BMO-PUL viscosity was observed when compared to that of native pullulan. The presence of free hydroxyl groups has been linked to swelling capacity; therefore, the attachment of the hydrophobic chain onto the pullulan backbone has reduced the presence of free hydroxyl groups, resulting in a decrease in the polymer aqueous viscosity [55]. This is in line with the findings of Lapčík Jr. et al. in their study on hyaluronic acid alkylation utilising various alkyl chain lengths [56,57].

A nanoprecipitation technique was utilised to formulate nanocarriers from BMO-PUL with varying DG and concentrations. The characteristics of the formulated nanoparticles are illustrated in Table 4; the diameter of the nanoformulations determined by DLS was in the range 125–185 nm, with DI values around 0.2 and negative ζ potentials ranged from −23 to −34 mV (recommending stable and narrowly dispersed nanoparticles) in agreement with the literature on the formulation of nanocarriers prepared from hydrophobically grafted pullulan acetate via nanoprecipitation, having the Z-av. less than 200 nm with negative ζ potential values [58]. The influence of the polymer concentration with varying DG used in the preparation of the nanoparticles was investigated, and an increase in the hydrodynamic diameter of the nanoparticles was observed to be directly proportional to the polymer concentration. However, an inverse proportion between the hydrodynamic diameter and the DG of BMO-PUL was recorded, which is parallel with the results discussed in the literature on the formulation of nanoparticles from amphiphilically modified pullulan [21,22].

**Figure 8:**
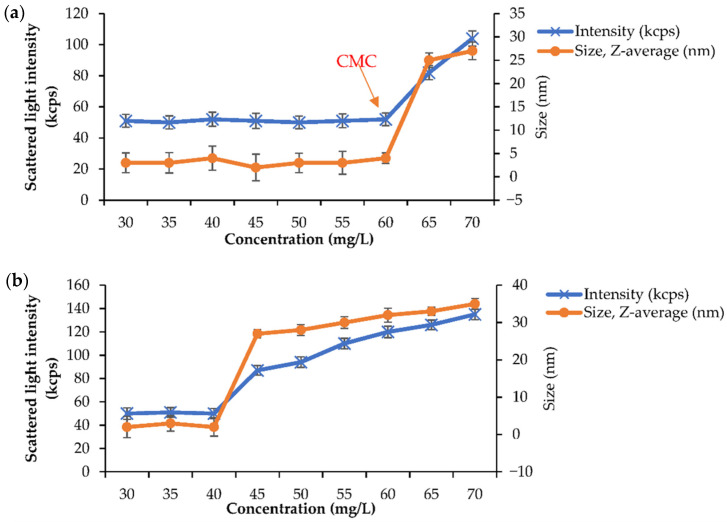
Scattering light intensity as a function of the concentration (range 30–70 mg/L) for BMO-PUL derivatives with different DG: (**a**) 12.6% and (**b**) 33.8% (*n* = 3; ± SD).

To investigate the CMC, a BMO-PUL aq. solution of varying concentrations (30–70 mg/L) in deionised H_2_O was prepared, and the analysis was performed following the methods described in the literature [59]. The scattered light intensity values as a function of BMO-PUL conc. are presented in Figure 8. Below the CMC, the scattering intensity values were approximately constant owing to that of deionised H_2_O. However, as the conc. increased above the CMC, a significant increase in the scattered light intensity, along with the nanoparticles/micelles size, owing to the augmentation of micelle formation, was observed, yielding the approximate CMC values of 60 mg/L and 40 mg/L for BMO-PUL with DG of 12.6% (Figure 8a) and 33.8% (Figure 8b), respectively. The values obtained in this study were found to be relatively consistent with the value reported by Chen et al. for an amphiphilic pullulan derivative [47]. Having low CMC values has been reported to lead to several advantages, including lower toxicity and greater micelles stability. Moreover, it has been noticed that the CMC values were also found to be inversely proportional to the Mw and the DG of BMO-PUL, as per the data reported in the literature [59].

**Figure 9:**
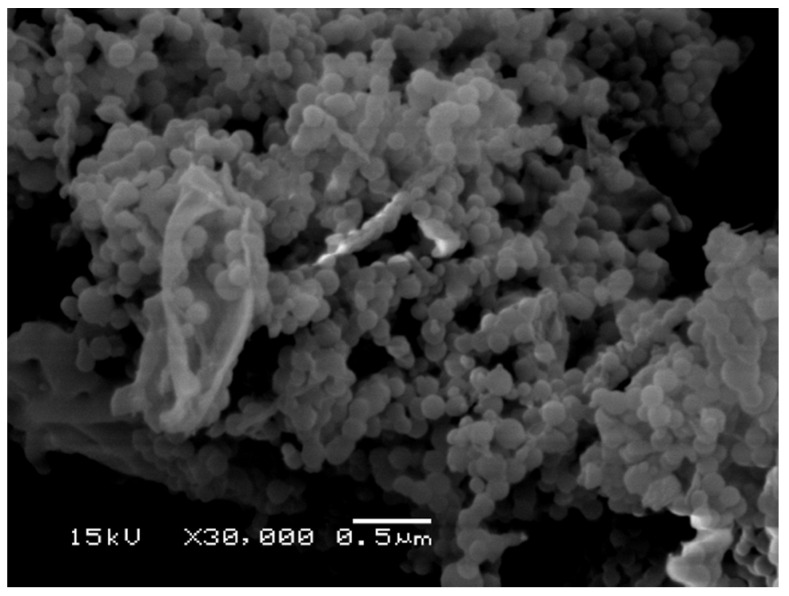
SEM micrograph of lyophilised BMO-PUL nanoparticles (Bar: 0.5 µm).

Microscopy investigation of the nanocarriers was performed by SEM, and a typical micrograph is illustrated in Figure 9, showing that the nanocarriers with submicron diameters exhibit close-to-spherical morphology, similar to the results described by Lee et al. [58]. A closely packed arrangement of BMO-PUL nanoparticles observed in the SEM image is attributed to the drying process during sample preparation [21].

In general, nanoparticles formulated from hydrophobically grafted materials have been more widely investigated with hydrophobic drugs, where high loading degree values (DL) have been reported compared to hydrophilic solutes [60,61,62]. In our study, a small water-soluble whitening agent, α-arbutin (Mw ~272 Da, Log P −1.49) [63], that is known to possess inadequate skin permeability was utilised as a model therapeutic to explore the loading ability and release profiles of BMO-PUL nanoparticles [33,34]. α-arbutin was loaded by formulation via nanoprecipitation, and the DL (as determined by RP-HPLC) was calculated utilising Equation (2); values of 16.1 ± 2.2% were obtained.

**Figure 10:**
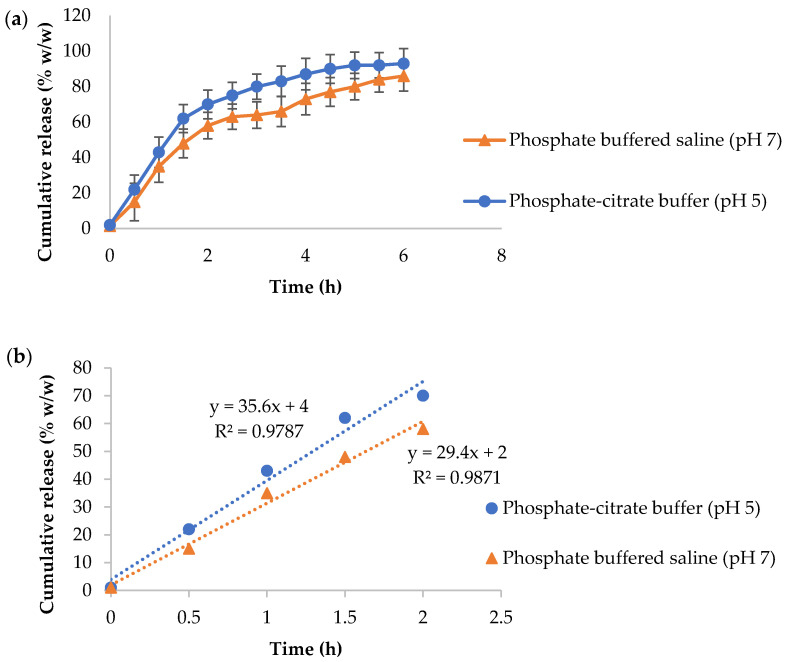
(**a**) Release profiles (0–6 h) and (**b**) linear regression profiles (0–2 h) of α-arbutin release from BMO-PUL nanocarriers (2 mg/mL) in either phosphate-citrate buffer (pH 5) or phosphate-buffered saline (pH 7) medium (*n* = 3; ± SD).

Rationalised by the fact that skin pH ranges between 4 and 7, α-arbutin released profiles were investigated in two different buffers, namely phosphate-citrate buffer (PCB, pH 5) and phosphate-buffered saline (PBS, pH 7) [64]. A similar pattern can be witnessed in both buffered solutions: a relatively fast discharge in the initial stage (0–2 h), continued by an incremental discharge for the following several hours (Figure 10a). This may be described by an initial discharge of adsorbed α-arbutin on the nanocarriers surface, prior to that entrapped within the matrix. A slightly faster release of α-arbutin was observed in mildly acidic buffer (PCB) compared to that of neutral pH (PBS), as shown from the slope of the line following linear regression (Figure 10b). This is likely due to fact that α-arbutin is a weak base (pKa ~10) that ionises more readily at acidic pH as compared to neutral, which facilitates its dissolution and release from the matrix of nanoparticles [65].

The data on drug release were fitted to known kinetic models (such as zero-order, first-order, Higuchi, Korsmeyer-Peppas, and Hixson-Crowell), and the R^2^ (squared correlation coefficient) values are summarised in Table 5. From the findings, the release of α-arbutin can be best described by a first-order model, with the rate being concentration-dependent [66]. The following is a quick overview of the other kinetic models that were tested: the Higuchi model describes drug release from insoluble and porous matrix based on Fickian diffusion. Korsmeyer-Peppas discusses simultaneous drug release processes from a polymeric structure, such as matrix dissolution and swelling. The Hixson-Crowell model depicts drug release from a system with a change in the particle or tablet surface area and diameter. Zero-order defines a system in which drug release is constant and independent of the concentration [67,68,69,70].

**Figure 11:**
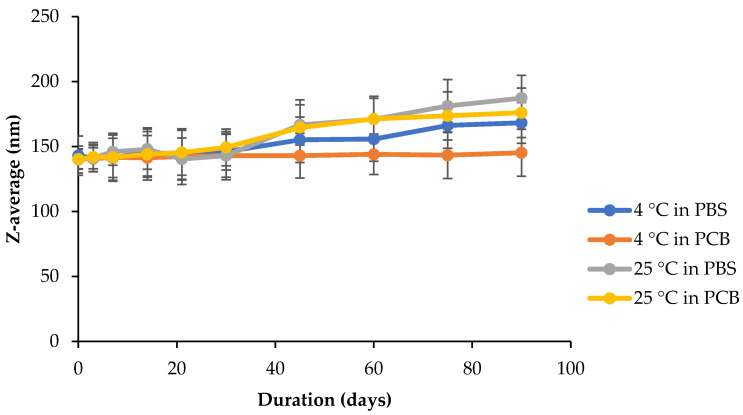
Hydrodynamic diameter of α-arbutin-loaded BMO-PUL NPs dispersed (2 mg/mL) in either phosphate-citrate buffer (pH 5) or phosphate-buffered saline (pH 7) at varying temperatures (4 or 25 °C) for 90 days (*n* = 3; ± SD).

The prospect of using nanoformulations as drug delivery vehicles is strongly linked to their size stability, which may impact their cytotoxicity and efficiency [71]. BMO-PUL nanoformulations were therefore examined for their stability at two pH values, phosphate-citrate buffer (pH 5) and phosphate-buffered saline (pH 7), at either 4 or 25 °C. Any possible changes in the nanoparticles diameter over 90 days were monitored, and the results are illustrated in Figure 11. Although the size continued to remain below 200 nm over the whole duration of the studies, incubation at 25 °C in both buffers witnessed a high increase in the nanoparticle diameters. In general, incubation at 4 °C induced only a small variation in the diameter, with almost no noticeable change, as was observed when incubated in PCB (indicating good stability, as evidenced by their ζ potentials in Table 4), as opposed to that of PBS. A high temperature is capable of increasing the kinetic movements of nanoparticles, which will ultimately promote collisions between them, which would increase their possibility to form bigger aggregates by van der Waals forces [72].

**Figure 12:**
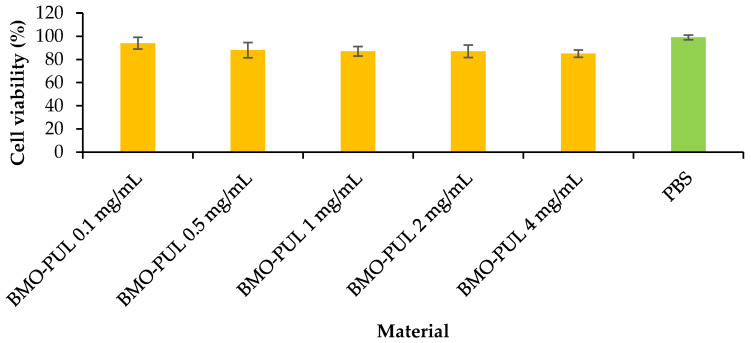
HaCaT cell viability data for α-arbutin-loaded BMO-PUL (concentration range 0.1–4 mg/mL) for 24 h (MTT assay; PBS as a control; *n* = 3; ± SD).

**Figure 13:**
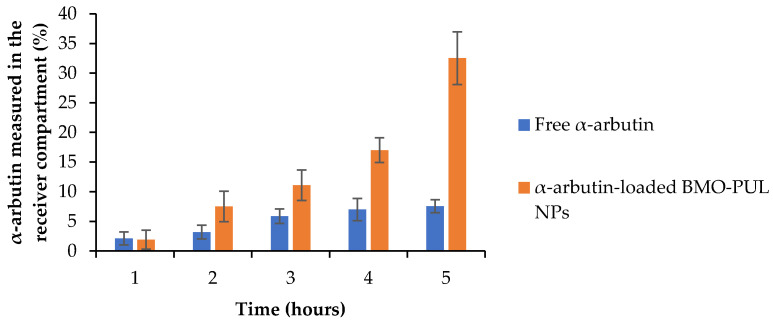
α-arbutin translocation (either as free model active or loaded within BMO-PUL NPs) across HaCaT cell monolayers (*n* = 5, ± SD).

The lack of cytotoxicity of the α-arbutin-loaded nanoparticles was evaluated in vitro at varying concentrations on HaCaT cells utilising an MTT assay (Figure 12). From the data (Figure 13), an increase in conc. did not initiate a significant reduction in cell viability (with more than 85% cell viability over the whole concentration range) following incubation for 24 h. This is in line with earlier findings that reported the lack of toxicity for nanoparticles formulated from hydrophobically grafted polysaccharides with short-chain alkylglycerols in various drug delivery applications [21,31].

Studies of the ability of BMO-PUL nanocarriers to increase the drug permeations through the HaCaT cell monolayers were done employing a modified Transwell model system. This system represents a reliable model for the permeability testing of percutaneous preparations, as it simulates the structure and stratification of normal human skin [39,73]. The concentration of loaded BMO-PUL NPs was calculated as equivalent to 0.1 mg of free α-arbutin. The results of the translocation of α-arbutin either as a free form or loaded within the NPs (Figure 13) showed after 5 h a four-fold increase in the α-arbutin amount transported as a nanoformulation compared to free α-arbutin.

The calculated permeability coefficients (P_app_) are presented in Table 6 and illustrate clearly a significantly higher translocation of α-arbutin across the membrane model when loaded within BMO-PUL NPs. The P_app_ values reported here were notably higher than those appearing in the literature, using a similar membrane model for FITC-dextran (Mw ~40 kDa), a high molecular weight hydrophilic solute (permeability coefficients of about 0.25–2.4 × 10^−8^), likely due to a significant difference in their molecular weight (α-arbutin Mw ~272 Da) [39]. The results presented in this work were considerably higher than earlier studies that investigated the permeation-enhancing effect of hydrophobically grafted guar gum nanocarriers (using the same Transwell-type HaCaT model), with only a two-fold increase in arbutin permeation reported [74]. This might be owing to the smaller size of the nanocarriers and the higher degree of grafting reported in this work. The increased permeation observed is not a result of any subsidiary toxicity to cells, as no significant cytotoxicity was observed (even at 4 mg/mL) in the results of the MTT assay (Figure 12; ≥85% of the cells are alive following 24 h of incubation with BMO-PUL NPs). Although the precise mechanisms have not been completely clarified, it can be presumed that these hydrophobically grafted materials may loosen the lipid tight packing and decrease the barrier resistance, leading eventually to enhanced permeation of solutes across the skin [75].

## 4. Future Directions and Limitations

With encouraging results obtained in the in vitro studies presented, future works need to concentrate on in vivo models, with several techniques such as micro-dialysis and tape stripping [76,77] outlined. These in vivo studies would offer the benefit of using a physiologically and metabolically active system; however, the necessity for radiolabelled material to allow accurate results and the difficulty in detecting the early absorption phase might represent significant limitations. Both animal and human skin can be used, with the latter preferred to minimise cross-species extrapolation; however, there are substantial ethical implications. The Organisation for Economic Cooperation and Development (OECD) guideline no. 428 addressed the standard principles for testing substances in vivo, such as the correct application of the test material, detection and quantification, etc. [78] Although rat skin is most commonly used, absorption tends to be overestimated due to its greater permeability compared to human skin. Other species such as pigs and monkeys may be closer to human absorption, but the costs are far greater [79].

## 5. Conclusions

Hydrophobically grafted pullulan (BMO-PUL) derivatives were successfully synthesised (DG 5–64%) under aq. alkaline conditions and subsequently formulated into narrowly dispersed nanoparticles (125–185 nm size) via nanoprecipitation. The prepared nanoparticles showed good colloidal stability at pH 5 and 7, with an α-arbutin loading degree of around 16%. A slightly faster α-arbutin release was observed under acidic conditions (compared to neutral pH), and the release profiles can be best described by a first-order model. No significant toxicity against the HaCaT cells was observed following incubation for 24 h with varying concentrations of BMO-PUL nanoparticles (0.1–4 mg/mL), and the translocation of α-arbutin through HaCaT cell monolayers was enhanced four-fold when the active was nanoformulated with BMO-PUL. Overall, the results indicated that BMO-PUL nanocarriers have adequate properties that would justify further research and optimisation of their potential for use in percutaneous drug delivery applications.

## Figures and Tables

**Table 1:** A summary of the thermal analysis results for BMO-PUL derivatives with different degrees of substitution (*n* = 3; ± SD).

Material (DG %)	H_2_O Evaporation		Decomposition		Tg (°C)
	DTG Peak (°C)	Mass Loss (%)	DTG Peak (°C)	Mass Loss (%)	
Native pullulan	80.9 ± 4.4	6.52 ± 1.9	288.5 ± 3.2	77.17 ± 6.8	21.3 ± 0.8
BMO-PUL (5.8%)	102.3 ± 4.8	5.32 ± 1.4	340.5 ± 4.9	78.02 ± 4.1	24.9 ± 0.7
BMO-PUL (12.6%)	104.5 ± 7.7	3.59 ± 0.9	349.2 ± 5.2	85.50 ± 5.9	26.7 ± 1.1
BMO-PUL (33.8%)	108.8 ± 5.1	2.93 ± 1.0	358.8 ± 3.6	88.67 ± 7.8	30.1 ± 1.2

**Table 2:** Estimated Mw of the BMO-PUL derivatives (*n* = 3; ± SD).

Material	Mn ± SD	Mw ± SD	DI ± SD
PUL (Mw 100 kDa)	77,121 ± 828	101,191 ± 923	1.31 ± 1.11
BMO-PUL (DG 12.6%)	86,236 ± 970	117,007 ± 911	1.36 ± 0.94
BMO-PUL (DG 33.8%)	99,991 ± 829	136,526 ± 931	1.37 ± 1.12

**Table 3:** BMO-PUL derivatives viscosity data (*n* = 3; ± SD).

Material	Viscosity η (cp)
Pullulan	1.82 ± 0.1
BMO-PUL (DG 12.6%)	1.58 ± 0.1
BMO-PUL (DG 33.8%)	1.41 ± 0.1

**Table 4:** BMO-PUL nanoformulation characteristics at various conc. and DG (*n* = 3; ± SD).

DG (%)	Polymer Conc. (mg/mL)	Diameter (nm)	DI	ζ Potential (mV)
12.6	1	143 ± 12	0.18 ± 0.07	−25.5 ± 3.0
	5	155 ± 16	0.17 ± 0.09	−23.3 ± 2.3
	10	182 ± 18	0.12 ± 0.06	−28.9 ± 4.1
33.8	1	136 ± 15	0.19 ± 0.05	−25.7 ± 1.9
	5	145 ± 19	0.12 ± 0.11	−23.7 ± 2.3
	10	173 ± 18	0.13 ± 0.09	−34.2 ± 4.2
47.4	1	125 ± 13	0.14 ± 0.11	−26.7 ± 5.2
	5	139 ± 12	0.18 ± 0.04	−28.6 ± 3.2
	10	158 ± 19	0.20 ± 0.07	−29.3 ± 4.1

**Table 5:** Quality of the fit (expressed as squared correlation coefficient, R^2^) for different kinetic models describing the release of α-arbutin from BMO-PUL nanoparticles.

pH Medium	Zero-Order	First-Order	Higuchi	Korsmeyer-Peppas	Hixson-Crowell
5	0.7986	0.9804	0.9489	0.3864	0.9384
7	0.8746	0.9829	0.9737	0.45	0.9641

**Table 6:** Experimental permeability coefficients (P_app_) of BMO-PUL nanoparticles through HaCaT cell monolayers at 5 h incubation time (*n* = 5; ± SD).

Material	P_app_ 5 h (cm/s)
Free α-arbutin	(1.29 ± 0.26) × 10^−6^
α-arbutin-loaded BMO-PUL NPs	(5.55 ± 0.14) × 10^−6^

## Data Availability

The raw data required to reproduce these findings can be shared upon request.

## Supplementary Materials

The following is available online at https://www.mdpi.com/article/10.3390/polym13172852/s1, Figure S1: ^13^C-NMR spectrum of 2-(butoxymethyl)oxirane.
