# Supplementary figures and images for: Hydrophobically Grafted Pullulan Nanocarriers for Percutaneous Delivery: Preparation and Preliminary In Vitro Characterisation
Source: Polymers (Basel). 2021 Aug 25;13(17):2852. doi: 10.3390/polym13172852 (PMC8434112; doi:10.3390/polym13172852)

## Supplementary Materials

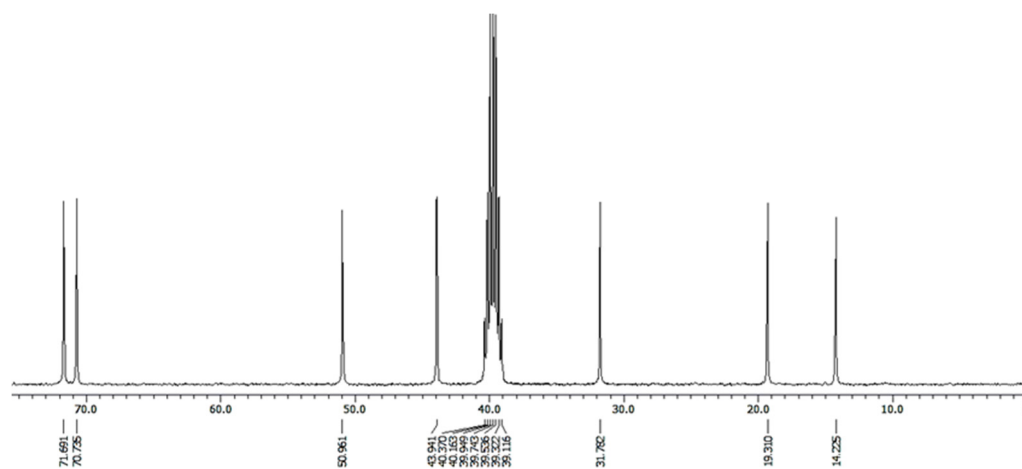

**Figure S1.**  $^{13}\text{C}$ -NMR spectrum of 2-(butoxymethyl)oxirane.

Supplement: Supplementary file 1 [file polymers-13-02852-s001.zip › polymers-1347393-supplementary.pdf]
